# Farmer’s knowledge and suggested approaches for controlling aflatoxin contamination of raw milk in Pakistan

**DOI:** 10.3389/fmicb.2022.980105

**Published:** 2022-10-20

**Authors:** Agha Waqar Yunus, Johanna Frida Lindahl, Zahid Anwar, Aman Ullah, Mohammed Nawaz Mohammed Ibrahim

**Affiliations:** ^1^Animal Sciences Institute, National Agricultural Research Center, Islamabad, Pakistan; ^2^Department of Animal Genomics and Biotechnology, PARC Institute of Advanced Studies in Agriculture, Islamabad, Pakistan; ^3^International Livestock Research Institute, Hanoi, Vietnam; ^4^Department of Clinical Sciences, Swedish University of Agricultural Sciences, Uppsala, Sweden; ^5^Department of Medical Biochemistry and Microbiology, Uppsala University, Uppsala, Sweden; ^6^International Livestock Research Institute, ILRI-Pakistan, Islamabad, Pakistan

**Keywords:** aflatoxin, awareness, dairy farmer, milk, mitigation, willingness to pay

## Abstract

Monitoring of aflatoxin levels in milk is often complicated in developing countries due to the dominance of informal markets channeling milk in raw form. Farmer’s awareness and voluntary participation in aflatoxin mitigation can be critical in such scenarios. Therefore, the present study was conducted to understand the perceptions of dairy farmers about aflatoxins and link it with aflatoxin mitigation programs on milk in Pakistan. Information was collected from 450 peri-urban dairy farmers in seven cities using questionnaires. Majority (77.9%) of the farmers were aware of the negative impact of moldy feed on animal health. However, only 40.6% of the farmers were aware of the transferability of the toxins from moldy feed to milk. The farmers had almost no awareness of aflatoxins as 95% never heard of the term. After receiving an onsite briefing on effects of the toxin on animal and human health, and its transferability to milk, 98.3% farmers showed willingness to buy aflatoxin-safe feedstuffs, while 88.5% showed willingness to control aflatoxin in milk. Around half of the farmers considered aflatoxin control programs as affordable. On average, farmers agreed to pay 10.1% higher price for aflatoxin certified oilseed cakes. Availability of feedstuffs certified of low aflatoxin content was suggested by 22% of the participants as the critical step in reducing aflatoxins in milk. Other important suggestions included; subsidy on quality feeds (18%), raising awareness (18%), and legislation and monitoring (16%). The present results suggest that the current practice of milk monitoring in the country can yield desirable results only if it is coupled with feed certification programs ensuing availability of aflatoxin-safe feeds. Further, awareness can positively impact participation of producers in aflatoxin control programs. In this regard, awareness about effects of aflatoxins on animal health was found to be a more powerful trigger of voluntary control compared with the awareness of the toxin’s transferability to milk.

## Introduction

Aflatoxins are mycotoxins produced by some *Aspergillus* spp. which commonly contaminate agricultural produce including grains, legumes, nuts, and various animal feedstuffs. Aflatoxins are classed as group 1 carcinogens and contribute to the global burden of hepatocellular carcinoma (IARC, 2012). In addition, aflatoxins suppress immunity and growth rate in both humans and animals (Reddy et al., 2010; Yunus et al., 2011; Atherstone et al., 2016). Among various types of aflatoxins, aflatoxin B_1_ has highest carcinogenic potency. After consumption by lactating animals, aflatoxin B_1_ is excreted as aflatoxin M_1_ (AFM_1_) in milk, potentially posing a health hazard to the consumers of milk and milk products (Yunus et al., 2011; IARC, 2012). Studies conducted in different low and middle-income countries have invariably found milk to be frequently contaminated with AFM_1_ to different extents, particularly in sub-Saharan Africa (Lindahl et al., 2018; Kagera et al., 2019; Kemboi et al., 2020), and Asia (Iqbal et al., 2022; Salari et al., 2022). In Pakistan, various surveys indicate that raw milk from peri-urban dairy farms is heavily contaminated with AFM_1_ and more than 90% of samples may exceed the CODEX maximum tolerable level of 500 ng/l (Muhammad et al., 2010; Yunus et al., 2019).

With growing awareness about food safety and more international trade, an increasing number of countries are establishing regulatory limits for mycotoxins (FAO, 2004). Aflatoxins are the most commonly regulated mycotoxins but the regulations vary between countries which may hamper trade and economic development (Wu and Guclu, 2012; Sirma et al., 2018). Similarly, the legislative levels of AFM_1_ allowed in milk vary, with most countries adopting the CODEX Alimentarius limit of 500 ng/l, which is the same as US FDA, while the EU and some other countries allowing only 50 ng/l (Kemboi et al., 2020). In case of Pakistan, control of aflatoxins and other mycotoxins have been a neglected area. The studies conducted to date in the country yielded different results, reporting mean AFM_1_ levels as low as 46 ng/l (Iqbal et al., 2011) to as high as 17,380 ng/l (Muhammad et al., 2010). These studies differed in sampling area, sampling season, and importantly also in the AFM_1_ quantification methodology. Based on the recently published literature, it may be generalized that more than 50% raw milk samples in major Pakistani cities exceed the 500 ng/l limit (Aslam et al., 2016; Asghar et al., 2018; Akbar et al., 2019; Yunus et al., 2019). However, processed milk has lower AFM_1_ levels than raw milk and has been found to generally comply with the 500 ng AFM_1_/L limit (Yunus et al., 2019; Iqbal et al., 2022). Further, the AFM_1_ contamination increases in winter (Hussain, 2009; Akbar et al., 2019; Yunus et al., 2019). In our own earlier study (Yunus et al., 2020), the mean AFM_1_ levels in peri-urban farms in different provincial capitals were found to be 3,185 ng/l during winter months, much higher than the recommended limit. Due to this situation, federal and some provincial governments have recently introduced legislation on aflatoxin levels in foods. For instance, amendment number 2 in standard PS-5344-2016 Pakistan Standards and Quality Control Authority, and Punjab Pure Food Regulations 2018 limit the maximum AFM_1_ in milk to 500 ng/l. These legislative measures have been effective in controlling the levels of the toxin in processed milk. However, as aforementioned, the AFM_1_ levels continue to be high in raw milk, which constitutes 95% of the total marketed milk in Pakistan (FAO, 2011).

While legislative limits for aflatoxins is the most common measure taken by governments, there are often problems with the implementation. In developing countries, where much of the milk is marketed in raw form, monitoring of AFM_1_ is complicated due to involvement of numerous small-scale producers and poor traceability. Furthermore, forceful implementation of standards can result in food security issues where high quality produce is available in limited quantities (Sirma et al., 2018). Therefore, aflatoxin mitigation programs should focus on voluntary participation of farmers by increasing awareness and giving them suitable options for improving milk quality. This is of particular relevance to the situation in Pakistan where literacy rate is low (GOP, 2016a). Although there has been an increase in the number of well-educated persons investing in the dairy sector in the recent past, the majority of farmers are still smallholders with low education. Therefore, majority of the farmers are expected to have little or no knowledge of emerging hazards like aflatoxin residues in milk. Support to this hypothesis comes from recent studies conducted in Kenya. Despite being a country with a history of aflatoxicosis incidences and much media attention toward aflatoxins, it has been found that dairy farmers in Kenya have low to medium level of knowledge about aflatoxins and the potential health risks of contaminated milk (Kagera et al., 2019; Kuboka et al., 2019). In light of above, the present study was therefore aimed at assessing the awareness of aflatoxins among dairy farmers in peri-urban setups of Pakistan, and identifying the critical measures that may be taken to assist farmers in voluntary control of AFM_1_ in milk. The findings of this study can be used as a baseline for evaluating interventions to improve awareness and to understand the mitigation options perceived feasible by farmers.

## Materials and methods

### Sample size and sampling sites

**Figure 1:**
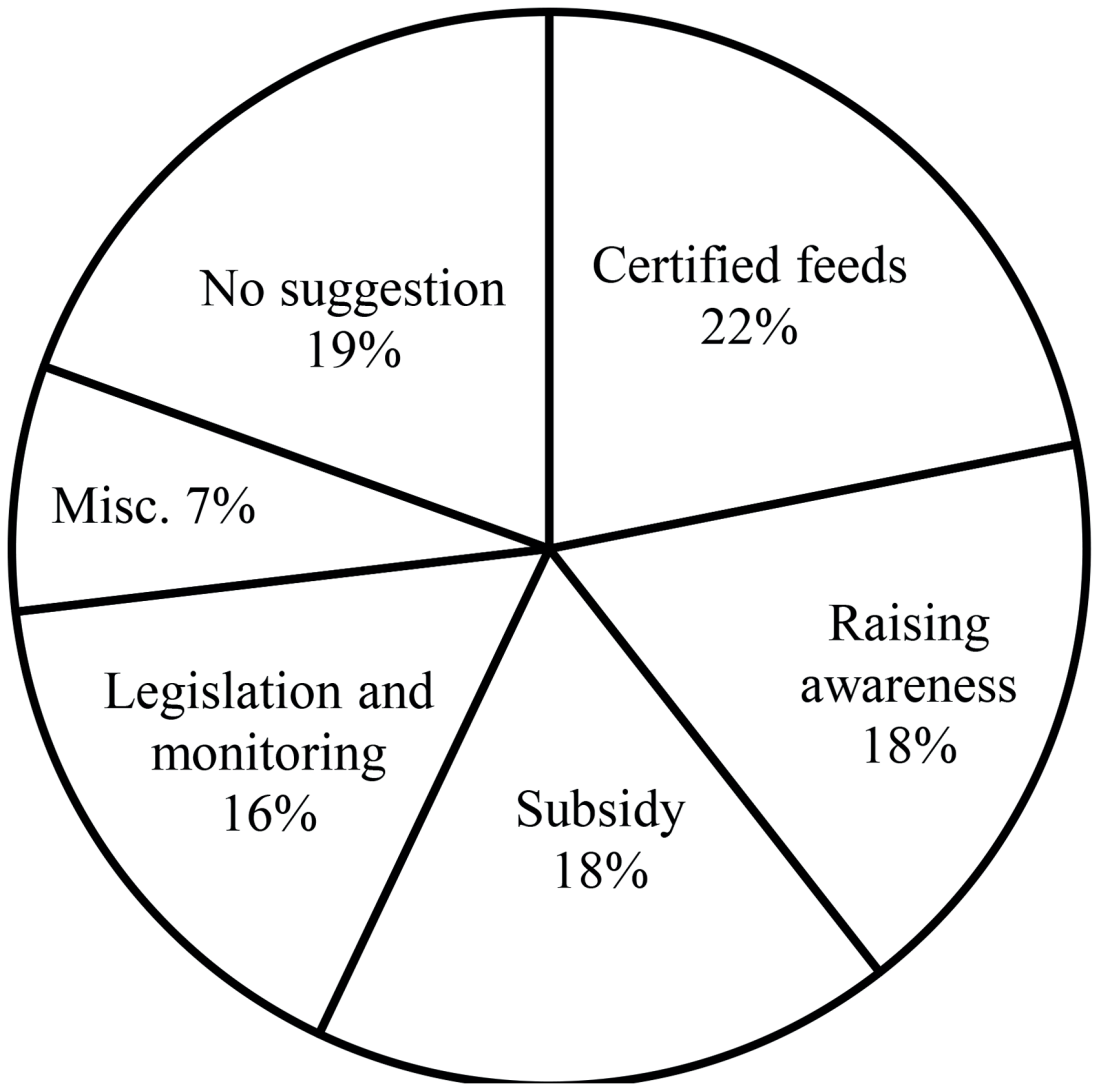
Farmer’s suggestions regarding aflatoxin control.

The current study was conducted through a cross-sectional survey of peri-urban dairy farmers in the federal and all the provincial capitals of Pakistan during October to mid-December 2016. This study was conducted on farmers who contributed milk and feed samples for another study on aflatoxin contamination of milk, and we have described the sample size calculations in detail previously (Yunus et al., 2020). In brief, a total sample size of 384 dairy farms was calculated with a proposed prevalence of 50% incidence of AFM_1_, 95% confidence interval, and 80% power/precision of the study. The number of farms were then increased by approximately 10% to accommodate refusal and sampling losses. Sample size for each city was calculated using stratified random sampling using probability proportional to size (PPS), and keeping in view the livestock population data and expert opinion of the Provincial Livestock and Dairy Development Departments. Finally, the study included 450 dairy farms all across Pakistan (Islamabad = 75, Karachi = 70, Lahore = 90, Quetta 50, Peshawar = 75, Muzaffarabad = 50, and Gilgit = 40; Figure 1). For this study, a peri-urban dairy farm was defined as the one located within the boundaries of the city districts and having a minimum of 2 dairy animals (cows or buffalo) intended for milk sale. In case of Gilgit, this criterion was relaxed to a minimum of one animal owing to small herd sizes in the city.

### Data collection

The data were collected during direct interviews with dairy farmers using a questionnaire. The questionnaire comprised of demographic questions, herd composition, farmers’ knowledge, attitude, and practices (KAP) about fungal contamination of animal feeds and aflatoxins, effects of aflatoxins on animal and human health. The demographic questions included information about the education and farming experience. Most of the questions were closed end questions where the response was divided into various categories. The questionnaire divided education level of the farmers into primary school (up to 5 years), secondary school (10 years), graduate (14 years), and postgraduate (16 years), and no education at all. Dairy farming experience consisted of five categories including up to 2, 3–5, 6–10, 11–25, and >25 years.

Some questions were designed to judge the awareness of aflatoxins, their sources and effects on animal and human health. Different questions were also asked regarding the effects of moldy feed on animal health and diseases observed by the farmers, awareness of aflatoxins and their transfer to milk, and impacts of aflatoxins on animal and human health. Lastly, each farmer was briefed about aflatoxins, their sources, carryover to milk, and the effects on animal and human health. After briefing, farmers were inquired if they desire to reduce aflatoxin in the dairy feeds and the milk they produce, and how much they were ready to pay for feed certified to be low in aflatoxins (in rupees). This was an open question, where the farmer could give any sum they wanted. Finally, suggestions were sought about aflatoxin control program based on the experiences of the dairy farmers.

Data were collected on paper questionnaires during field visits to the farmers on their dairy farms. The questions were explained to farmers in their local languages by the investigators and answers were marked. Each questionnaire was assigned a unique ID for data entry and analysis.

### Statistical analysis

The data for various variables used in the study are presented as proportions for categorical variables or means for continuous variables. Means were compared using analysis of variance (ANOVA) and least significant differences (LSD) in IBM SPSS Statistics 20 (IBM Corp., Armonk, New York, NY, United States, 2011). Proportions of categorical variables were compared using Chi square statistics. Data for willingness to voluntarily control aflatoxins in milk were compared between cities using logistic regression in STATA 14.2 (STATACorp LLC, College Station, TX, USA).

## Results

### Level of education and farming experience

The data regarding education and experience of farmers are presented in Tables 1, 2, respectively. Only 16.9% farmers had graduation or higher degrees. One third of the participants had attended secondary school (10 years education), and 22.9% had no education at all. Although the farming experience ranged from 1 year for a new farmer to over 25 years, about 73% of the farmers had been in dairy farming for over 10 years.

**Table 1:** Education of peri-urban dairy farmers in different Pakistani cities.

City	*n*	Percentage (%) of farmers				
		None (%)	Primary (%)	Secondary (%)	Graduate (%)	Postgraduate (%)
Islamabad	74	23.0	25.7	36.5	12.2	2.7
Lahore	86	37.2	20.9	33.7	8.1	-
Muzaffarabad	50	16.0	26.0	30.0	20.0	8.0
Karachi	51	7.8	37.3	43.1	11.8	-
Peshawar	73	35.6	24.7	31.5	6.8	1.4
Quetta	46	19.6	28.3	32.6	8.7	10.9
Gilgit	39	-	33.3	20.5	30.8	15.4
Total	419	22.9	27.0	33.2	12.6	4.3

None, no education; primary, 5 years; secondary, 10 years; graduate, 14 years; postgraduate, 16 years.

**Table 2:** Experience of peri-urban dairy farmers in different Pakistani cities.

City	*n*	Percentage (%) of farmers				
		≤2 years (%)	3–5 years (%)	6–10 years (%)	11–25 years (%)	>25 years (%)
Islamabad	74	29.7	12.2	17.6	18.9	21.6
Lahore	89	1.1	2.2	4.5	18.0	74.2
Muzaffarabad	50	-	6.0	18.0	42.0	34.0
Karachi	67	4.5	10.4	10.4	46.3	28.4
Peshawar	71	4.2	14.1	8.5	22.5	50.7
Quetta	48	6.3	12.5	14.6	31.3	35.4
Gilgit	39	-	5.1	2.6	10.3	82.1
Total	438	7.3	8.9	10.7	26.6	46.3

### Knowledge about moldy feeds

The data on responses of the farmers to questions on molds and aflatoxins in feed are presented in Table 3. Over 75% of the respondents agreed to the fact that moldy feed may damage animal health. The farmers who were aware of the risks of molds had more (*p* = 0.02) milking animals (mean 50.6 milking animals, standard error (SE) 3.6), compared to the farmers who were not aware (mean 24.4, SE 5.0). Out of the farmers agreeing to the negative health effects of moldy feed, over 75% rated these effects to be medium to severe in nature. In follow up open-ended questions on the diseases caused by moldy feed, 28.3% of the respondents rated diarrhea as the major disease caused by consumption of moldy feed. Other diseases reported by farmers included mastitis (26.1%), toxemia (10.3%), feed refusal (9.9%), miscellaneous digestive problems including bloat, constipation, and ceased rumination (5.5%), fever (3.7%), lethargy and weakness (2.6%), liver toxicity (1.1%), reproductive disorders (0.7%), and miscellaneous problems including low milk production, skin diseases, and death (3.3%). Some respondents (6.6%) did not know any disease caused by moldy feed. Regarding the transferability of toxins from moldy feed to milk, only 40.6% of the farmers answered that these are transferred to milk. Out of these farmers, 43.8% considered milk of such animals to pose serious to medium health hazard for consumers.

**Table 3:** Knowledge of farmers about moldy feed and its negative health effects.

Questions^*^	Responses					
	Yes (%)	No (%)	Do not know (%)	Serious (%)	Medium (%)	Minor (%)
Do you think moldy feed can damage health of animals?	77.9	8.9	13.2	-	-	-
If yes, how severe are the health risks to animals?	-	-	8.7	46.2	29.2	15.8
Do you think there are toxins in moldy feed that can be transferred to milk consumers?	40.6	19.9	39.5	-	-	-
If yes, how severe are health risks associated with consuming milk of animals fed moldy feed?	-	-	30.0	15.5	28.3	26.2

* Question number 2 and 4 were asked from only those farmers which responded positively to question 1 and 3.

### Awareness of aflatoxins and willingness to mitigate

Dairy farmers had almost no knowledge about aflatoxins as 95.1% of the surveyed farmers never heard of the term ‘aflatoxin’ (Table 4). The farmers who had heard about aflatoxins had more (*p* < 0.001) milking animals (mean 102.2, SE 23.3), compared to the farmers who did not know about aflatoxins (mean 41.6, SE 2.9). Out of the farmers who previously heard about aflatoxins, 70.6% considered these to have serious to medium negative effects on animal health. However, 52.9% of these farmers did not know about transferability of aflatoxins to milk. Only 47.6% of the farmers who previously heard about aflatoxins (i.e., only 10 farmers) tried to control aflatoxins during their dairy farming career. In this regard, excluding cottonseed cake and waste bread from dairy rations were practiced by 40%, while use of toxin binders was practiced by 30% of these farmers.

**Table 4:** Awareness and willingness to control aflatoxin in milk.

City	Respondents aware about AFM_1_ (%)	Post briefing		
		Willingness to buy aflatoxin free feed (%)	Willingness to voluntarily lower AFM_1_ in milk (%)	Suitable extra cost for certified oilseed cakes (Rs/kg)^1^
Islamabad	4.0	100.0	97.3^a^	1.6 ± 1.4^d^
Lahore	2.5	98.8	97.4^a^	3.5 ± 2.4^c^
Muzaffarabad	6.0	100.0	98.0^a^	4.7 ± 2.5^b^
Karachi	14.7	98.3	54.2^c^	0.5 ± 1.0^e^
Peshawar	0.0	98.6	91.5^a^	3.5 ± 2.4^c^
Quetta	2.1	91.5	76.6^b^	4.6 ± 3.6^b^
Gilgit	7.7	100.0	100.0^a^	7.2 ± 4.4^a^
Total	4.9	98.3	88.5	3.3 ± 3.1

1 Extra cost respondents were ready to pay for oilseed cakes certified for aflatoxin levels ± standard deviation.

^abc^Means bearing different superscript differ significantly within a column at *p* < 0.01.

When the respondents were briefed about aflatoxins, 98.3% expressed willingness to control the toxin in the dairy feedstuffs they use. However, a lesser percentage, i.e., 88.5% of the farmers expressed willingness to also voluntarily control AFM_1_ in the milk they produce.

There was no significant (*p* = 0.85) difference between the different education categories as to their willingness to control aflatoxin levels in dairy feeds, with 97.9, 98.2, 98.5% in the three less educated categories, and 100% in the more educated categories. There was however a significant influence of experience, as farmers with less experience were least willing to control the level of aflatoxins in feed. Among the farmers with up to 2 and 5 years’ experience, only 93.8 and 91.7%, respectively were willing to control aflatoxins; lower (*p* = 0.002) than more experienced farmers (100% in farmers with up to 7.5 and 17.5 years’ experience, and 99.0% in farmers with longer experience). The education and experience of farmers willing or not willing to voluntary control aflatoxin in milk after receiving briefing was not different (*p* > 0.60).

### Aflatoxin mitigation and affordability

Overall, the farmers showed willingness to pay Rs 3.3/kg (US$ 0.032) extra for oilseed cakes certified to have lower aflatoxin contamination (Table 4). In this regard, respondents from Gilgit were ready to pay the highest (*p* < 0.001) per kg cost, i.e., Rs 7.2/kg. The respondents from Karachi were least (*p =* 0.019) willing to pay extra cost for feedstuffs certified to be free of aflatoxins. Overall, only 37.2% of the farmers considered implementing aflatoxin control program as unaffordable (Table 5). In case of the respondents from Karachi, however, 94.6% considered aflatoxin control programs to be unaffordable.

**Table 5:** Respondent’s opinion (%) about affordability of aflatoxin control in milk.

Questions	Unaffordable (%)	Affordable (%)	Do not know (%)
Islamabad	35.4	53.8	10.8
Lahore	5.4	89.2	5.4
Muzaffarabad	48.9	37.8	13.3
Karachi	94.6	1.8	3.6
Peshawar	5.9	61.8	32.4
Quetta	30.0	20.0	50.0
Gilgit	5.1	71.8	23.1
Total	37.2	47.0	15.9

Respondents were also asked about their opinion about potential control strategies of aflatoxin control. The responses of farmers in this regard are presented in Figure 1. The main suggestions in order of preference were certification of feedstuffs, raising awareness among stakeholders, price control and subsidies on good quality feed and relevant medicines, legislation on aflatoxin control and monitoring of feed sellers. Farmers from various cities responded differently in this regard. While 85.3% of the respondents from Gilgit were in favor of raising awareness among stakeholders, 68.8 and 55.1% of the farmers in Lahore and Peshawar, respectively were in favor of feed certification and legislation. While 37% of the farmers in Quetta suggested subsidies on good quality feed and medicines, no farmer suggested this in Gilgit and Muzafarrabad. Over 40% of the farmers in Islamabad and Karachi gave no suggestion in this regard.

## Discussion

### Level of education and farming experience

This study shows low degree of understanding of aflatoxins among peri-urban dairy farmers in Pakistan. The presently reported 77.1% literacy rate among dairy farmers was higher than the national average of 60% (males) during the survey year (GOP, 2016a). This is probably because the survey was undertaken in peri-urban areas of provincial capital cities where literacy rate is expected to be higher than that in the villages. This notion is supported by the study of Rajper (2006) who reported lower literacy rate among dairy farmers in less developed areas of Naushahro Feroze district in the province of Sindh, Pakistan. The present figures on education status in combination with the observation that almost half of the famers had over 25 years’ experience indicate that most of them were not new to this profession. Thus, dairy farming in general can be regarded as a family profession in the country. This trend was evident across Pakistan, except in Islamabad (*p* = 0.004) where 29.7% of the farmers were new to dairy farming with an experience of less than 2 years. This implies that dairy farming is becoming a popular and profitable business in the capital city where many new people are turning in to this business.

Contrary to the present data, Ajmal et al. (2015) reported 62% of dairy farmers to have 12 years of education, and 54% to have less than 11 years of farming experience in five districts of Punjab province. These authors however also reported almost 100% dairy farmers to have attained primary school which is quite inconsistent with 71% literacy rate in the province of Punjab for the year 2014 (GOP, 2016b). The figures obtained in the study of Ajmal et al. may be explained by the small sample size and possible lack of representativeness. Overall, the education level among dairy farmers in Southeast Asia is often reflective of the national averages. In neighboring India for instance, Rathod et al. (2011) reported 45% education among dairy farmers in Karnataka, while Deka et al. (2020) reported 63.5% farmers to have less than 10 years of education in Assam and Bihar, India.

### Knowledge about moldy feeds

Present data indicate that farmers had some degree of awareness of the detrimental effects of moldy feed on animal health as over two thirds of them agreed to the negative impact of moldy feed on animal health. Like the present results, Nguyen et al. (2018) reported that in Son La province of Vietnam, only 1.1% farmers heard of aflatoxins but 55% did regard moldy maize to be harmful for humans and cattle. The present results and the studies conducted in Vietnam show that farmers may not be familiar with the names of fungal toxins but they do have some degree of realization of the negative health effects of moldy feed. However, this awareness can be regarded as conventional wisdom lacking scientific grounds as most of the farmers related the effects of moldy feed with diseases which are not actually caused by it. Thus in Kenya, where many projects on aflatoxin control have been undertaken, farmers were found to report abdominal pain, heartburn, vomiting, diarrhea and even typhoid as results of moldy maize consumption (Kiama et al., 2016). It is yet interesting to note that almost 40% of farmers both in the present study and in the study of Kiama et al., agreed that milk of animals fed on moldy feed can have negative effects on consumers. Respondents in the study of Kiama et al. (2016) even reported molds to transfer from feed to milk. Also, in the study of Nguyen et al. (2018) in Vietnam, 62.6% respondents realized that eating meat from animals fed moldy feed is not safe but 84.1% respondents consumed meat of such animals. These figures point out lack of proper awareness campaigns in the developing countries, but also the fact that food may be consumed even when not considered completely safe.

### Aflatoxin awareness

There is a dearth of information on awareness of aflatoxins among dairy farmers in Asia. A survey on maize growers in Vietnam indicates that awareness of aflatoxins in farmers is generally low and varies between zero to 23.3% in six provinces (Lee et al., 2017; Nguyen et al., 2018). The data on dairy farms from developing countries in Africa support some of the findings of the present study. In this regard, Kiama et al. (2016) found that 89% of the participant farmers from different risk groups in Kenya regarded moldy feed to exert negative effects on animal health. However, awareness of aflatoxins among dairy farmers in different countries of Africa has been reported to be different. In this regard, over 75% dairy farmers in Tanzania (Ayo et al., 2018), and over 90% of feed processors and dairy farmers in Rwanda were found to have never heard about aflatoxins (Nishimwe et al., 2019). Although, awareness of aflatoxins was found to be correlated with level of education in Tanzania, the study conducted in Rwanda reported no such correlations. Even 26% of the farmers in the study in Rwanda had university degrees - much higher than the presently noted 14.9%. These data imply that education of farmers alone may not be connected to awareness of aflatoxins. Rather, community education and national awareness programs are required to increase farmer awareness. Support to this notion comes from the reports published from some African countries where both the national and international organizations have been working on aflatoxin mitigation and increasing awareness. Thus, 80% of the peri-urban dairy farmers in Kenya were reported in a recent study to have heard of aflatoxins, with 55% having right information about the toxin (Kagera et al., 2019). Similarly, 85% of surveyed crop growers in Congo were found to have heard of aflatoxins, with 50% having sufficient knowledge of their negative effects on animal health and transfer to milk (Udomkun et al., 2018). In the later study, awareness of aflatoxins was correlated with education level of crop farmers in Congo. It seems that implementation of various projects on aflatoxin mitigation in some regions of Africa, as also noted by Nyangi et al. (2016), resulted in changes in awareness of aflatoxins. Farmers from regions in Nigeria with aflatoxin campaigns had higher aflatoxin awareness compared with regions where no such campaigns were launched (Johnson et al., 2018), indicating the importance of these campaigns. Since aflatoxins are invisible, and can only be detected by laboratories, it is important to increase knowledge with awareness campaigns. As there has not been any effort from government or non-governmental organizations on this issue in Pakistan, the awareness of aflatoxins is low. Furthermore, the aflatoxin control in milk in Pakistan is only being observed by corporate milk processors and their suppliers. As peri-urban farmers in Pakistan sell milk directly to consumers, there is an absence of impetus to understand the toxin. This factor coupled with absence of awareness programs on aflatoxin would result in lack of correlation of awareness on the toxin with education.

A few reports on peanut farmers and consumers in neighboring India also support our conclusions regarding aflatoxin awareness. In this regard, Kumar and Popat (2010) concluded that socioeconomic and psychological characteristics including education, caste, farm size, social participation, extension participation, market orientation, economic motivation have positive and significant associations with knowledge of aflatoxin in peanut farmers. Kumar and Popat in their study also found that extension staff and traders had a good understanding of the problem and of the importance of managing aflatoxin contamination but farmers did not.

Contrary to our conclusions, Yeole and Deshmukh (2013a, 2013b) reported differences in education level of respondents having different awareness of aflatoxin in two surveys of consumers and farmers in Maharashtra, India. In the first and second survey, 36 and 60% of the respondents, respectively were found aware about aflatoxins. In both the surveys, literacy rate among respondents was 100% but 39% respondents in the first while 64% in the second survey were graduates or postgraduates. In the words of Yeole and Deshmukh, the “*civilized status”* of respondents affected the differences in awareness of aflatoxins in their study. A detailed look at their data reveals that 34% respondents in the first survey while only 16% in the second were agriculturists while rest of the respondents belonged to other profession categories including jobs, business, or miscellaneous. This shows that people engaged in agriculture have lower awareness of contemporary issues compared to people engaged in other professions. Overall, this also supports our inference that dedicated campaigns on aflatoxin education are needed to increase awareness of aflatoxins.

### Willingness to voluntarily control aflatoxins

To the best of our knowledge, this is first attempt in which post briefing willingness to control aflatoxin levels in milk and feed were judged. More farmers were willing to voluntarily control aflatoxin in feed than the farmers willing to control aflatoxin in milk. It is interesting to note that experienced dairy farmers were more willing to control aflatoxin in feed. We have previously reported that the famers in the city of Karachi, with highest aflatoxin awareness, did not use cottonseed cake (Yunus et al., 2020) which has been reported as the main aflatoxin contaminated ingredient in dairy rations in Pakistan (Hussain, 2009; Yunus et al., 2020). However, this practice could be a result of frequent cases of cattle poisoning associated with the use of cottonseed cake as also reported by us previously (Yunus et al., 2015). These reports and the present results indicate that farmers could be more concerned with health of cattle than the transferability of aflatoxin to milk. It appears from these results that the knowledge about negative impact of aflatoxins on animal health is a stronger trigger of paradigm shift compared with the knowledge about transferability of the toxin to milk. This could be because farmers are more concerned with the health of the dairy animals which are a source of income and livelihood. Therefore, awareness programs aimed at voluntary control of aflatoxin in milk should include briefing about negative effects of aflatoxins on health of the dairy animals and milk productivity. This inference is also supported by experience gained during efforts to control AFM_1_ in milk a decade ago by the Pakistani milk processing industry. The field teams involved in increasing awareness and reducing AFM_1_ in milk found that farmers started actual control of aflatoxin in dairy feeds when they became aware of its negative effects on animal health (Akhtar, n.d. personal communication).

### Aflatoxin mitigation and affordability

In the present study, farmers on an average were ready to pay Rs 3.3/kg (US$ 0.032/kg) more for feeds certified to have low aflatoxins. This amount is quite reasonable as it was 10.1% higher than the normal price of dairy feeds in the study year. The present study also highlighted presence of differences in cities regarding extra cost farmers consider suitable for aflatoxin safe feeds. In this regard, farmers in Gilgit were ready to pay highest cost which could be in part due to the higher literacy rate in Gilgit region (Rehman et al., 2015). As also shown in Table 1, the number of graduate respondents (42.6%) from Gilgit in the current study were higher than other cities. This figure was only 11.6% in Karachi, where farmers were least willing to spend extra money on certified feeds and also considered mitigation programs as unaffordable. Such responses are understandable because the feed inputs in Karachi are costlier compared with rest of the country (Afzal, 2008). Peri-urban dairy farming in Karachi, country’s largest metropolitan city, is unique as it is restricted to large cattle colonies established for this purpose and farmers pay for almost every farm input from farm rent to feed and veterinary support. Land availability is limited and crop farming for fodder production is not practical. At the same time, the profit margins are lower due to high disease burden, expensive transportation, and consumer’s limited affordability. Thus, the farmers in Karachi are already in a very competitive business environment and additional farm inputs like paying higher price for mycotoxin free feed are generally not welcome, as reflected in this study. This is despite the fact that these farmers are usually better aware of issues like mycotoxins than traditional livestock farmers in other livestock production systems.

A limitation of this study is that the data on willingness to pay were collected using an open question rather than a choice experiment. This number likely does not reflect what would be paid in reality. Earlier studies using choice card experiments found that poultry farmers in Nigeria (Johnson et al., 2020), and milk consumers in Kenya (Mtimet et al., 2015) were willing to pay more for feedstuffs and milk, respectively certified to be free of aflatoxins. One problem with assessing willingness to pay, is that these may not reflect the long-reality. Thus, in an experiment with maize for human consumption in Kenya, only a temporary willingness to pay premium price was found and that too after intensive marketing (Hoffmann et al., 2021). It is therefore, unlikely that farmers would actually be willing to pay as much as stated in this study.

Majority of the farmers in the country have small to medium herd sizes (Yunus and Hasan, 2021) and do not have access to feed analysis facilities. There is also no institution for certification of dairy feed regarding its quality. In these circumstances, it is not practical for the farmers to control aflatoxins in feed and milk even if there is a desire to do so. Therefore, the suggestions regarding feed certification and legislation are rational and need to be given priority in AFM_1_ mitigation programs.

## Conclusions and way forward

Studies conducted in other countries suggest that AFM_1_ daily exposure is usually lower than 1 ng/kg body weight (WHO, 2017). For instance, the average daily exposure to AFM_1_ from milk was found to be 0.3 to 1.0 ng/kg in Kenya (Ahlberg et al., 2018; Sirma et al., 2019). From these exposure levels, the overall cancer risk from milk was estimated to be less than 0.007 cases per 100,000 for all age groups. It has therefore been urged that the benefits of consuming milk outweigh the risks of developing cancer. Aside from the risk of cancer however, AFM_1_ has been suggested to cause stunted growth in 2.7% of children in Kenya (Ahlberg et al., 2018). If stunted growth due to AFM_1_ is proven, it would be alarming for Pakistan as the overall daily exposure to AFM_1_ through milk in certain urban areas of the country has been estimated to be 4 to 15 times higher than in Kenya (Jawaid et al., 2015; Yunus et al., 2019). Therefore, it is important to reduce the AFM_1_ exposure for the urban milk consumers of Pakistan.

Currently, some of the provincial governments in Pakistan are monitoring aflatoxins in milk. However, these efforts are restricted only to processed milk which has only 5% share in total milk marketed in the country. The milk processing companies were successful in complying with the aflatoxin limits by implementing thorough testing coupled with a pricing policy in which milk price for producer was paid inversely proportional to the toxin content. Such a policy is not practical in case of raw milk which has over 90% of the milk marketed share (Yunus and Hasan, 2021) and has very high levels of AFM_1_, especially from peri-urban dairy farms (Yunus et al., 2019, 2020). Monitoring and controlling raw milk is difficult because of the hurdles in traceability, involvement of small holders, and informal marketing channels. To improve the quality of the raw milk it is important to mobilize farmers for improvement of the milk quality. This study found that majority of the dairy farmers in Pakistan, like in other countries, have very low understanding of aflatoxins and their effects. It is apparent that when awareness of aflatoxins is low, voluntary control of the toxin in milk is not expected from the farmers. Therefore, it is important to invest in community mobilization programs through awareness. In this regard, this study highlights that the knowledge on effects of aflatoxins on animal health compared to the knowledge about its transferability to milk could more effectively influence farmer’s decision to control aflatoxins. As this fact has also been previously observed by field workers in Pakistan, this could be used as a game changer in awareness programs targeted on reducing AFM_1_ in raw milk.

This study further points out that any awareness campaign on aflatoxins has to be integrated with feed certification and appropriate legislation. Compared to monitoring of AFM_1_ levels in milk, a more practical approach to improve quality of raw milk marketed through informal channels would be to invest in certification of dairy feeds for aflatoxin content under one health slogan. This is comparatively easier to implement due to traceability of the stuff especially the commercial dairy feeds, and has a direct relationship with AFM_1_ levels in milk.

In surveys conducted in Kenya (Mtimet et al., 2015), and Pakistan (Abedullah et al., 2018), it was found that consumers are ready to pay premium price for aflatoxin free milk. These reports and the present data indicate that raising awareness among consumers of milk, besides the dairy farmers, could increase pace of mitigation programs on making milk safe. A long term and sustainable approach would however be to also educate consumers to use packaged milk which is not only traceable but is also marketed after laboratory testing in most cases.

## Data availability statement

The raw data supporting the conclusions of this article will be made available by the authors, without undue reservation.

## Ethics statement

The studies involving human participants were reviewed and approved by Technical Expert Committee, NARC and passed the institutional Research Management Cell (PRMC). Written informed consent for participation was not required for this study in accordance with the national legislation and the institutional requirements.

## Author contributions

AY and MI: prepared concept. AY and AU: finalized methodology. AY, AU, and ZA: collected data. AY, JL, and AU: analyzed data. AY: wrote original draft. AY and JL: edited and finalized manuscript. AY and MI: supervised field activities. AY and MI: acquired funds and administered project. All authors contributed to the article and approved the submitted version.

## Funding

This research was funded under the framework of collaborative research agreement between National Agricultural Research Center (NARC) and International Livestock Research Institute (ILRI) under the USAID funded Agriculture Innovation Program (AIP) for Pakistan led by CIMMYT, and also additionally supported by the CGIAR Initiative One Health, “Protecting human health through a One Health approach”. The findings of this study are the sole responsibility of NARC and ILRI, and do not necessarily reflect the views of USAID or United States Government.

## Conflict of interest

The authors declare that the research was conducted in the absence of any commercial or financial relationships that could be construed as a potential conflict of interest.

## Publisher’s note

All claims expressed in this article are solely those of the authors and do not necessarily represent those of their affiliated organizations, or those of the publisher, the editors and the reviewers. Any product that may be evaluated in this article, or claim that may be made by its manufacturer, is not guaranteed or endorsed by the publisher.
